# Impact of Upadacitinib Induction and Maintenance Therapy on Health-related Quality of Life, Fatigue, and Work Productivity in Patients with Moderately-to-severely Active Crohn’s Disease

**DOI:** 10.1093/ecco-jcc/jjae083

**Published:** 2024-06-05

**Authors:** Subrata Ghosh, Brian G Feagan, Rogério Serafim Parra, Susana Lopes, Adam Steinlauf, Yoichi Kakuta, Namita Joshi, Wan-Ju Lee, Ana P Lacerda, Qian Zhou, Si Xuan, Kristina Kligys, Nidhi Shukla, Edouard Louis

**Affiliations:** College of Medicine and Health and APC Microbiome Ireland, University College Cork, Cork, Ireland; Robarts Research Institute, Western University, London, ON, Canada; Alimentiv Inc., London, ON, Canada; Ribeirão Preto Medical School, University of São Paulo, São Paulo, Brazil; Centro Hospitalar e Universitário São João, Porto, Portugal; IBD Clinical Center, Mount Sinai Hospital, New York, NY, USA; Division of Gastroenterology, Tohoku University Graduate School of Medicine, Sendai, Japan; Health Economics & Outcomes Research, AbbVie Inc., North Chicago, IL, USA; Health Economics & Outcomes Research, AbbVie Inc., North Chicago, IL, USA; Health Economics & Outcomes Research, AbbVie Inc., North Chicago, IL, USA; Health Economics & Outcomes Research, AbbVie Inc., North Chicago, IL, USA; Health Economics & Outcomes Research, AbbVie Inc., North Chicago, IL, USA; Health Economics & Outcomes Research, AbbVie Inc., North Chicago, IL, USA; Health Economics & Outcomes Research, AbbVie Inc., North Chicago, IL, USA; Department of Gastroenterology, Centre Hospitalier Universitaire de Liège, Liège, Belgium

**Keywords:** Clinical trials

## Abstract

**Background and Aims:**

Quality of life in patients with active Crohn’s disease may be significantly reduced. We evaluated the effects of upadacitinib induction and maintenance therapy on fatigue, quality of life, and work productivity in the phase 3 trials U-EXCEL, U-EXCEED, and U-ENDURE.

**Methods:**

Clinical responders to upadacitinib 45 mg in U-EXCEL and U-EXCEED induction trials were re-randomised 1:1:1 to upadacitinib 30 mg, 15 mg, or placebo for 52 weeks of maintenance in U-ENDURE. Clinically meaningful improvements in Inflammatory Bowel Disease Questionnaire [IBDQ] response, IBDQ remission, Functional Assessment of Chronic Illness Therapy–Fatigue [FACIT-Fatigue], and Work Productivity and Activity Impairment were evaluated. Percentages of patients achieving clinically meaningful improvements were assessed at induction Weeks 4 and 12 and maintenance Week 52.

**Clinical Registration number:**

U-EXCEED induction trial [NCT03345836], U-EXCEL induction trial [NCT03345849], U-ENDURE maintenance trial [NCT03345823].

**Results:**

Analysis included 1021 and 502 patients assessed at induction and maintenance, respectively. In U-EXCEL, greater improvements [all *p* ≤ 0.001] in IBDQ response [71.0% vs 50.2%], IBDQ remission [44.2% vs 23.7%], and FACIT-Fatigue [42.0% vs 27.0%] were observed in upadacitinib-treated patients versus placebo at Week 4. Improvements in IBDQ response, IBDQ remission, and FACIT-Fatigue were similar or greater at Week 12. Clinically meaningful improvement in overall work impairment [52.1% vs 38.1%, *p* ≤ 0.05] was demonstrated at Week 12. Similar results were observed in U-EXCEED. Improvements were sustained through 52 weeks of upadacitinib maintenance treatment.

**Conclusions:**

In patients with active Crohn’s disease, upadacitinib treatment relative to placebo significantly improved fatigue, quality of life, and work productivity as early as Week 4. These effects were sustained through 52 weeks of maintenance.

## Graphical Abstract

**Graphical Abstract:**
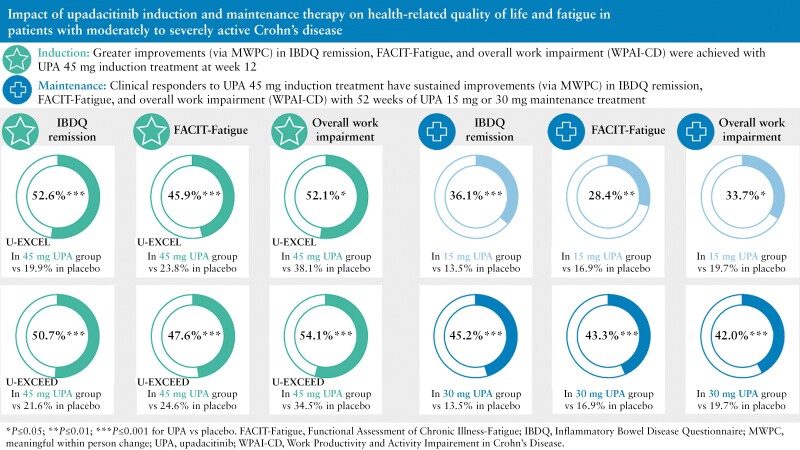

## 1. Introduction

Crohn’s disease [CD] is a chronic, progressive, and relapsing inflammatory bowel disease [IBD] affecting the gastrointestinal tract.^1^ Current treatment approaches focus on symptom control via early clinical response and clinical remission, inflammation control as measured by biomarkers, and endoscopic response with the ultimate goal of achieving mucosal healing.^2^ Available treatment options include immunomodulators, corticosteroids, and biologic agents; however, these do not always elicit or maintain clinical response and may induce adverse effects, indicating the need for additional therapeutic options.^3–7^

Health-related quality of life [HRQoL] is a multidimensional construct focusing on patients’ perceptions of their physical, psychological, and social functions.^8^ Patients with IBD report poor HRQoL and disability, fatigue, and work impairment.^9^ CD-related symptoms can severely affect patients’ HRQoL, including: physical function, such as fatigue, sleep quality, and pain; social function, such as loss of social satisfaction; psychological function, including depression and anxiety; and aspects of work productivity.^10–15^ Approximately half of patients with moderately-to-severely active CD have some degree of work impairment, with annual indirect costs from work impairment estimated at $29,524 per patient.^16^

Fatigue, a common, burdensome,^17^ yet largely unexplored^2^ symptom, is experienced by patients with CD^9^ irrespective of disease activity.^18^ It affects more than 80% of patients with active disease^19^ and is associated with poor HRQoL, more active disease, reduced work productivity, and higher rates of unemployment.^15,20,21^

Patient perspectives on fatigue, work productivity, and HRQoL are important, given the considerable burden fatigue poses on patients’ HRQoL,^22,23^ the potential lack of awareness among health care professionals of the importance of this symptom,^17^ and the relevance of HRQoL and patient-reported outcomes to patients’ acceptance and adherence to therapy.^24^ The Selecting Therapeutic Targets in Inflammatory Bowel Disease [STRIDE-II] initiative identified restoration of HRQoL as a crucial long-term treatment goal, independent of achieving clinical remission, normalisation of biomarkers of inflammation, and endoscopic/histological mucosal healing.^2^

The phase 3 induction and maintenance trials [U-EXCEL, U-EXCEED, and U-ENDURE] demonstrated the efficacy and safety of upadacitinib in patients with moderately-to-severely active CD with regard to achievement of clinical remission and endoscopic response.^25^ In this analysis, we report the effects of upadacitinib on disease-specific and generic HRQoL measures, fatigue, and work productivity/daily activity in patients participating in the U-EXCEL, U-EXCEED, and U-ENDURE trials.

## 2. Materials and Methods

### 2.1. Study design

U-EXCEL [NCT03345849] and U-EXCEED [NCT03345836] were phase 3, multicentre, randomised, double-blind, placebo-controlled, induction trials of the efficacy and safety of upadacitinib in patients with moderately-to-severely active CD who had an inadequate response or intolerance to one or more conventional and/or biologic therapies, as previously described.^25^ Both U-EXCEL and U-EXCEED trials consisted of a 12-week induction period and a 12-week extended treatment period for patients who did not achieve clinical response at the end of the induction period. U-EXCEED also included an open-label, upadacitinib induction arm, to achieve sufficient clinical responders for the subsequent maintenance trial.

U-ENDURE [NCT03345823] was a 52-week, phase 3, multicentre, randomised, double-blind, placebo-controlled, maintenance trial investigating the efficacy and safety of upadacitinib for patients with CD who achieved clinical response in U-EXCEL and U-EXCEED.

The trials were conducted at 277 sites in 43 countries globally.

### 2.2. Study cohort

As previously described,^25^ in the U-EXCEL and U-EXCEED induction trials, patients 18–75 years old were randomised 2:1 to receive oral upadacitinib 45 mg once daily [QD] or placebo QD.^25^ Moderately-to-severely active CD was defined as average daily stool frequency [SF] ≥ 4 and/or abdominal pain score [APS] ≥ 2, along with a Simple Endoscopic Score for CD [SES-CD; excluding the narrowing component subscore] ≥ 6 [≥ 4 for patients with isolated ileal disease], confirmed by a central reader. Patients had a diagnosis of CD for at least 3 months. A protocol-specific taper was initiated at Week 4 for patients receiving corticosteroids at baseline of induction treatment; the taper was continued at the beginning of the maintenance trial for patients who did not complete it during induction. A clinical response was defined as ≥ 30% decrease in average, daily, very soft or liquid SF and/or average daily APS, with neither value greater than at baseline. Patients with a clinical response following 12 weeks of upadacitinib 45 mg were enrolled in U-ENDURE and re-randomised 1:1:1 to receive upadacitinib 15 mg QD, upadacitinib 30 mg QD, or placebo QD for 52 weeks of maintenance treatment.

### 2.3. Patient-reported outcomes

To capture the potential benefits of upadacitinib treatment on HRQoL, fatigue, and work productivity, we assessed clinically meaningful improvements for several patient-reported outcome measures. The measures assessed included the Inflammatory Bowel Disease Questionnaire [IBDQ], Functional Assessment of Chronic Illness Therapy–Fatigue [FACIT-Fatigue], Short-Form Health Survey-36 version 2 [SF-36v2] Physical Component Summary [PCS] and Mental Component Summary [MCS], EuroQol 5 Dimension [EQ-5D], and Work Productivity and Activity Impairment in Crohn’s Disease [WPAI-CD]. Outcome measures were collected at induction Weeks 4 and 12 and maintenance Week 52 of the upadacitinib trials.

The IBDQ is a disease-specific instrument comprising 32 Likert-type questions measuring HRQoL.^26^ It covers four subdomains: bowel symptoms [eg, abdominal pain, loose stools], systemic symptoms [eg, sleep patterns, fatigue], emotional function [eg, irritability, anger, depression], and social function [eg, work attendance]. An increase of ≥ 16 points in IBDQ total score from baseline^27^ and a total score ≥ 170 points are considered IBDQ response and remission, respectively.^28^

The FACIT-Fatigue is a Likert-type questionnaire with 13 items measuring fatigue,^29,30^ and increases of ≥ 9-points from baseline to Weeks 4 or 12 of induction or Week 52 of maintenance were considered as meaningful within-person change [MWPC].^31^

The SF-36v2 is a generic HRQoL instrument with eight subscales measuring functioning [physical functioning, perception of general health, role limitations due to physical health challenges, bodily pain, vitality, role limitations due to emotional challenges, social role functioning, and mental health] and yielding PCS and MCS scores. Higher scores indicate better HRQoL.^32–34^ An increase of ≥ 4.1-points for PCS and ≥ 3.9-points for MCS between baseline and Weeks 4 or 12 of induction, respectively, or Week 52 of maintenance were considered to have met the MWPC criteria.^35^

EQ-5D is an instrument used for the evaluation of generic health status and HRQoL. Patients classify their health according to five dimensions [mobility, self-care, usual activities, pain/discomfort, anxiety/depression] at three different levels [no problems, some problems, unable/extreme problems] and rate their current health status on each dimension using a visual analogue scale [VAS] from 0 [worst imaginable health state] to 100 [best imaginable health state].^36,37^ An increase of ≥ 9.2 points in EQ-5D VAS from baseline to Weeks 4 or 12 of induction or Week 52 of maintenance was considered to constitute MWPC.^35,38^

The WPAI-CD is a disease-specific instrument assessing the impact of CD on work productivity and performance of daily activities.^39^ It comprises four domains, namely presenteeism [impairment while working], absenteeism [work time missed], overall work impairment, and activity impairment.^39^ A decrease of ≥ 6.1% in presenteeism, ≥ 6.5% in absenteeism, ≥ 7.3% in overall work impairment, and ≥ 8.5% in activity impairment were considered an MWPC.^40^

### 2.4. Statistical analyses

Analyses for U-EXCEL and U-EXCEED were conducted using the intention-to-treat population [ie, all randomised patients who received one or more doses of upadacitinib]. For U-ENDURE, analyses were performed on data from patients who achieved a clinical response to upadacitinib during induction.

Patient demographic and baseline characteristics were summarised with descriptive statistics. The proportions of patients who achieved clinically meaningful improvements [via MWPC] in IBDQ response, IBDQ remission, FACIT-Fatigue, SF-36v2 PCS, and SF-36v2 MCS from baseline to Weeks 4 and 12 of induction and Week 52 of maintenance were reported. Comparisons were made between the upadacitinib 45 mg and placebo groups in the U-EXCEL and U-EXCEED induction trials, and between the upadacitinib 15 mg or upadacitinib 30 mg and placebo groups in the U-ENDURE maintenance trial. Adjusted risk differences of upadacitinib compared with placebo, 95% confidence intervals, and *p*-values were calculated using the Cochran–Mantel–Haenszel test. Risk differences were adjusted for randomisation strata in the U-EXCEL and U-EXCEED induction trials (baseline corticosteroid use [yes or no], endoscopic disease severity [SES-CD < 15 or ≥ 15], and number of prior biologics with inadequate response or intolerance [0, 1, or > 1 for U-EXCEL; > 1 or ≤ 1 for U-EXCEED]), as well as in the U-ENDURE maintenance trial (prior induction population [failure or non-failure to biologics], APS/SF clinical remission status [yes or no], and endoscopic response [yes or no] at the end of induction). Calculations were based on non-responder imputation; multiple imputation was incorporated to handle missing data due to the COVID-19 pandemic.

To examine the relationship between clinical remission, endoscopic response, or corticosteroid-free remission and individual HRQoL measures, we compared HRQoL outcomes in upadacitinib-treated patients who achieved clinical remission, endoscopic response, or corticosteroid-free remission with those who did not achieve these pre-specified outcomes at induction Week 12, using the chi square test. Clinical remission was defined as Crohn’s Disease Activity Index [CDAI] < 150 at Weeks 4 and 12. Endoscopic response was defined as a decrease in SES-CD > 50% from baseline [or for patients with baseline SES-CD of 4, at least a 2-point reduction from baseline]. In patients taking corticosteroids at baseline, those who discontinued corticosteroid use for CD and achieved CDAI clinical remission at Week 12 were considered to have achieved corticosteroid-free remission. A similar analysis was conducted to evaluate the relationship between the proportion of patients who achieved clinically meaningful improvements in FACIT-Fatigue and general HRQoL measures of SF-36v2 PCS and MCS. All missing values were considered non-response.

### 2.5. Ethics statement

All three trials were conducted in accordance with the International Conference on Harmonization guidelines and the Declaration of Helsinki. The protocol was approved by an independent ethics committee or institutional review board at each trial site. Written informed consent was provided by all patients who took part in the trials.

## 3. Results

### 3.1. Patient baseline characteristics

A total of 1021 patients in U-EXCEL and U-EXCEED and 502 upadacitinib clinical responders in U-ENDURE were included in this analysis. Baseline characteristics and demographics were similar across treatment groups and have been previously reported.^25^

CD had a substantial negative impact on the patients enrolled in the upadacitinib trials, as demonstrated by baseline outcome scores [Table 1]. Mean IBDQ scores at baseline were below 130 [range: 117–122 across groups], which is indicative of severely active disease.^41^ Both SF-36v2 PCS and MCS baseline scores ranged from 38 to 40, which is lower than the average in healthy individuals [50 and 46, respectively].^42,43^ For FACIT-Fatigue, baseline scores ranged from 23 to 25, which is substantially below the general population average of 44.^44^ Mean EQ-5D VAS scores ranged from 49 to 53, which is lower than the average score [80] in the general population.^45^

**Table 1:** Demographics and baseline characteristics of patients in the induction and maintenance trials.^a^

Characteristic	U-EXCEL [12 weeks]		U-EXCEED [12 weeks]		U-ENDURE [52 weeks]		
	Placebo [*n* = 176]	Upadacitinib 45 mg [*n* = 350]	Placebo [*n* = 171]	Upadacitinib 45 mg [*n* = 324]	Placebo [*n* = 165]	Upadacitinib 15 mg [*n *= 169]	Upadacitinib 30 mg [*n *= 168]
**Male sex**	94 [53]	189 [54]	96 [56]	169 [52]	88 [53]	102 [60]	93 [55]
**Age** [years], mean [SD]	39.3 [13.6]	39.7 [13.7]	37.5 [12.1]	38.4 [13.7]	38.1 [13.0]	38.1 [13.5]	37.0 [13.3]
**Race**							
White	130 [74]	258 [74]	126 [74]	230 [71]	119 [72]	118 [70]	114 [68]
Black or African American	4 [2]	17 [5]	6 [4]	19 [6]	11 [7]	6 [4]	7 [4]
Asian	36 [20]	73 [21]	38 [22]	69 [21]	35 [21]	43 [25]	45 [27]
Other^b^	6 [3]	2 [1]	1 [1]	6 [2]	0	2 [1]	2 [1]
**IBDQ at baseline**, mean [SD]	118.0 [33.8]	122.0 [34.3]	117.2 [31.4]	120.5 [33.8]	117.3 [34.8]	121.8 [33.9]	120.1 [30.9]
**FACIT-Fatigue at baseline**, mean [SD]	23.5 [12.3]	24.5 [12.1]	23.8 [12.4]	23.5 [12.2]	22.9 [11.9]	24.9 [12.5]	23.2 [11.5]
**SF-36v2 PCS at baseline**, mean [SD]	39.6 [8.1]	39.1 [8.1]	38.4 [8.4]	38.4 [8.1]	37.6 [7.9]	39.4 [8.6]	38.6 [8.0]
**SF-36v2 MCS at baseline**, mean [SD]	37.8 [11.2]	39.2 [10.5]	38.8 [11.5]	39.6 [11.0]	38.6 [10.4]	39.1 [11.0]	39.3 [9.9]
**EQ-5D VAS at baseline**, mean [SD]	50.5 [21.2]	52.6 [20.7]	49.3 [20.8]	50.9 [21.3]	50.1 [20.9]	51.1 [21.2]	52.8 [18.5]
**WPAI-CD presenteeism^c^ at baseline**, mean [SD]	50.0 [25.2]	47.1 [23.6]	45.4 [22.9]	48.3 [23.8]	50.4 [24.6]	49.2 [25.4]	47.3 [22.3]
**WPAI-CD absenteeism^c^ at baseline**, mean [SD]	20.7 [27.4]	19.5 [28.9]	18.8. [29.6]	22.8 [30.7]	21.1 [26.9]	21.1 [29.9]	24.1 [33.3]
**WPAI-CD overall work impairment^c^ at baseline**, mean [SD]	58.1 [28.2]	55.6 [27.5]	54.5 [27.1]	58.6 [28.0]	59.1 [27.7]	57.6 [28.7]	58.8 [27.2]
**WPAI-CD activity impairment at baseline**, mean [SD]	56.4 [25.8]	55.4 [26.2]	58.4 [24.4]	56.4 [25.0]	58.7 [24.5]	55.6 [27.2]	56.0 [23.3]
**Body mass index**, mean [SD]	25.6 [7.0]	24.5 [6.0]	23.9 [6.2]	24.2 [6.0]	24.6 [6.6]	24.1 [6.0]	24.2 [6.6]
**Disease duration** [years], median [range]	5.7 [0.3, 46.3]	6.7 [0.1, 52.1]	9.8 [0.6, 46.1]	9.3 [0.5, 55.2]	7.6 [0.3, 48.7]	7.9 [0.3, 40.1]	7.2 [0.3, 44.9]
**Concomitant Crohn’s disease medications**							
Immunosuppressants	3 [2]	13 [4]	13 [8]	24 [7]	11 [7]	5 [3]	9 [5]
Steroids	64 [36]	126 [36]	60 [35]	108 [33]	61 [37]	63 [37]	63 [38]
**Previous treatments**							
Prior biologic failure	78 [44]	161 [46]	171 [100]	324 [100]	126 [76]	124 [73]	127 [76]
No prior biologic failure	98 [56]	189 [54]	0 [0]	0 [0]	39 [24]	45 [27]	41 [24]
**Previous biologic failures**							
1	28 [36]	58 [36]	68 [40]	126 [39]	52 [41]	52 [42]	43 [34]
2	24 [31]	52 [32]	55 [32]	92 [28]	32 [25]	31 [25]	35 [28]
≥ 3	26 [33]	51 [32]	48 [28]	106 [33]	42 [33]	41 [33]	49 [39]

EQ-5D VAS, EuroQol 5 Dimension visual analogue scale; FACIT-Fatigue, Functional Assessment of Chronic Illness Therapy–Fatigue; IBDQ, Inflammatory Bowel Disease Questionnaire; MCS, Mental Component Summary; PCS, Physical Component Summary; SD, standard deviation; SF-36v2, Short-Form Health Survey-36 version 2; WPAI-CD, Work Productivity and Activity Impairment in Crohn’s Disease.

^a^From Loftus EV, Jr, Panes J, Lacerda AP, *et al*., Upadacitinib Induction and Maintenance Therapy for Crohn’s Disease, *N Engl J Med* 2023;**388(21)**:1966–80, copyright © 2023 Massachusetts Medical Society. Reprinted with permission from Massachusetts Medical Society.

^b^‘Other’ included patients who identified as American Indian/Alaska Native, Native Hawaiian or other Pacific Islander, or multiple races.

^c^Reported only for patients who were employed at baseline. Data are *n* [%], unless otherwise specified.

### 3.2. IBDQ response and remission

**Figure 1:**
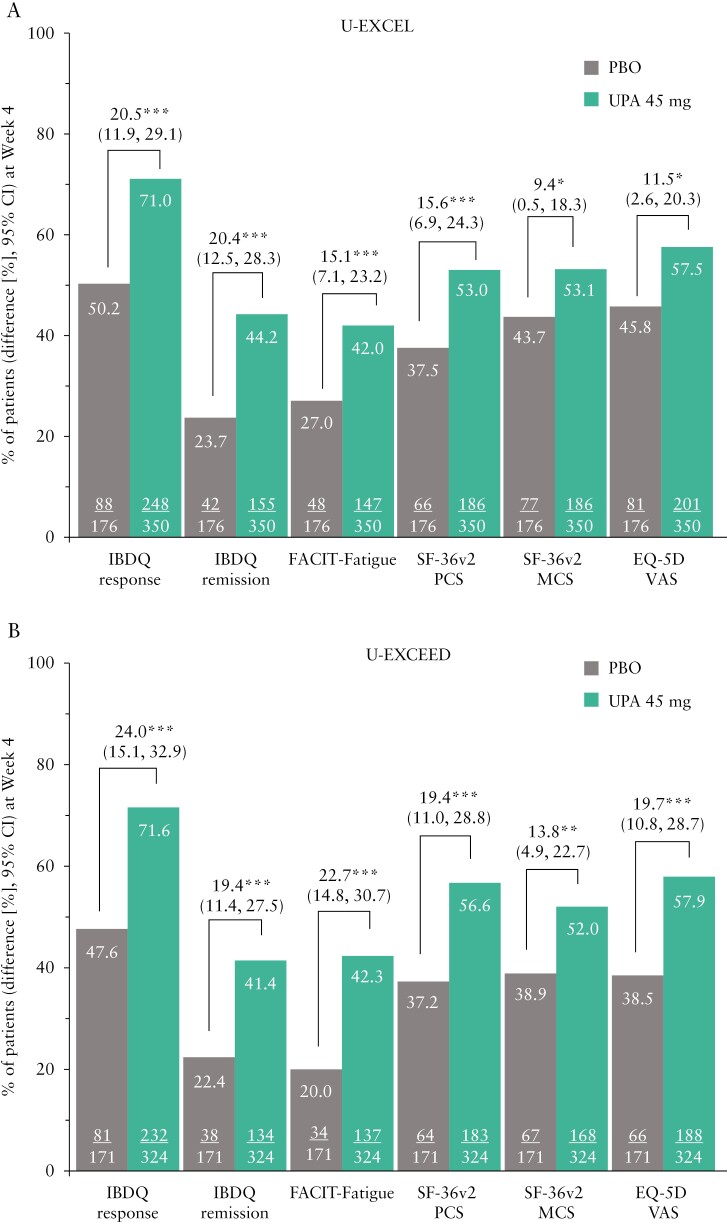

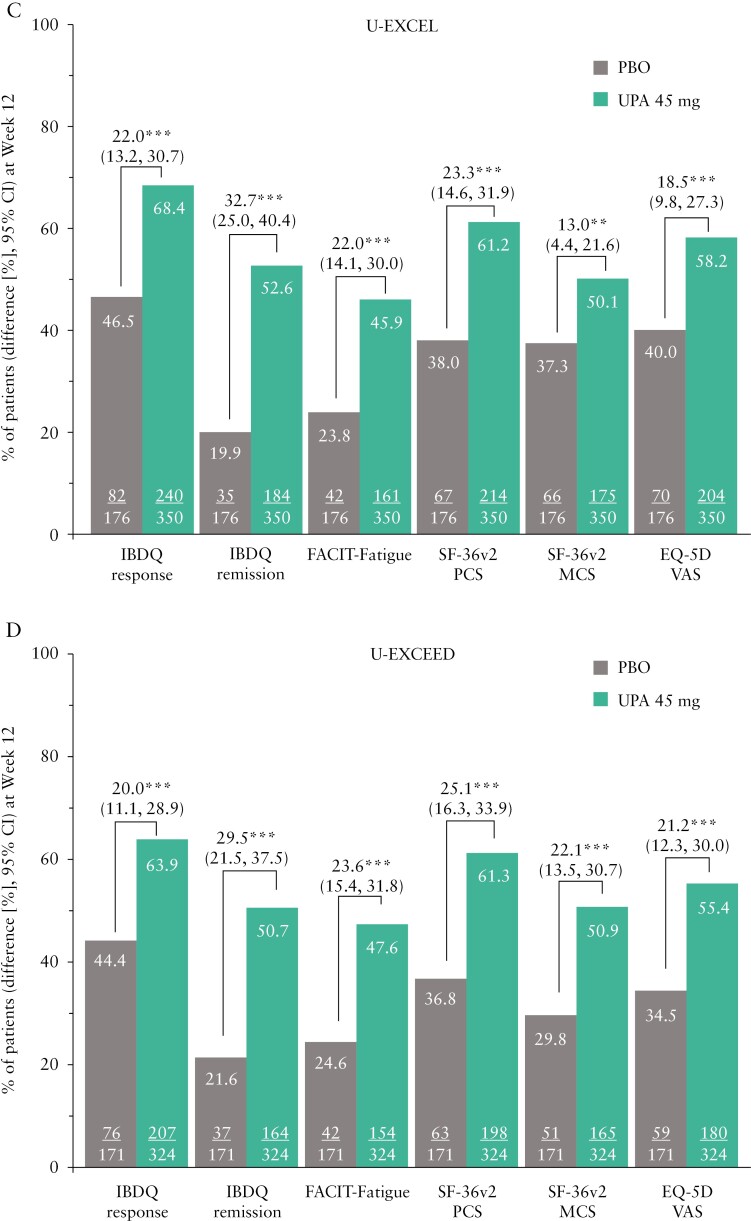
Percentage of patients reporting clinically meaningful improvements in IBDQ response, IBDQ remission, FACIT-Fatigue, SF-36 PCS, SF-36v2 MCS, and EQ-5D at [A, B] Week 4 and [C, D] Week 12 of the U-EXCEL and U-EXCEED induction trials; *p ≤ 0.05; **p ≤ 0.01; ***p ≤ 0.001 for upadacitinib versus placebo. An increase of ≥ 16 points in IBDQ total score from baseline and a total score ≥ 170 points were considered IBDQ response and remission, respectively. An increase of ≥ 9-points in FACIT-Fatigue, ≥ 4.1-points in SF-36v2 PCS, ≥ 3.9-points, SF-36v2-MCS, and ≥ 9.2-points in EQ-5D VAS from baseline was considered an MWPC. CI, confidence interval; EQ-5D VAS, EuroQol 5 Dimension Visual Analogue Scale; FACIT-Fatigue, Functional Assessment of Chronic Illness–Fatigue; IBDQ, Inflammatory Bowel Disease Questionnaire; MCS, Mental Component Summary; MWPC, meaningful within-person change; PBO, placebo; PCS, Physical Component Summary; SF-36v2, Short-Form Health Survey-36 version 2; UPA, upadacitinib.

**Figure 2:**
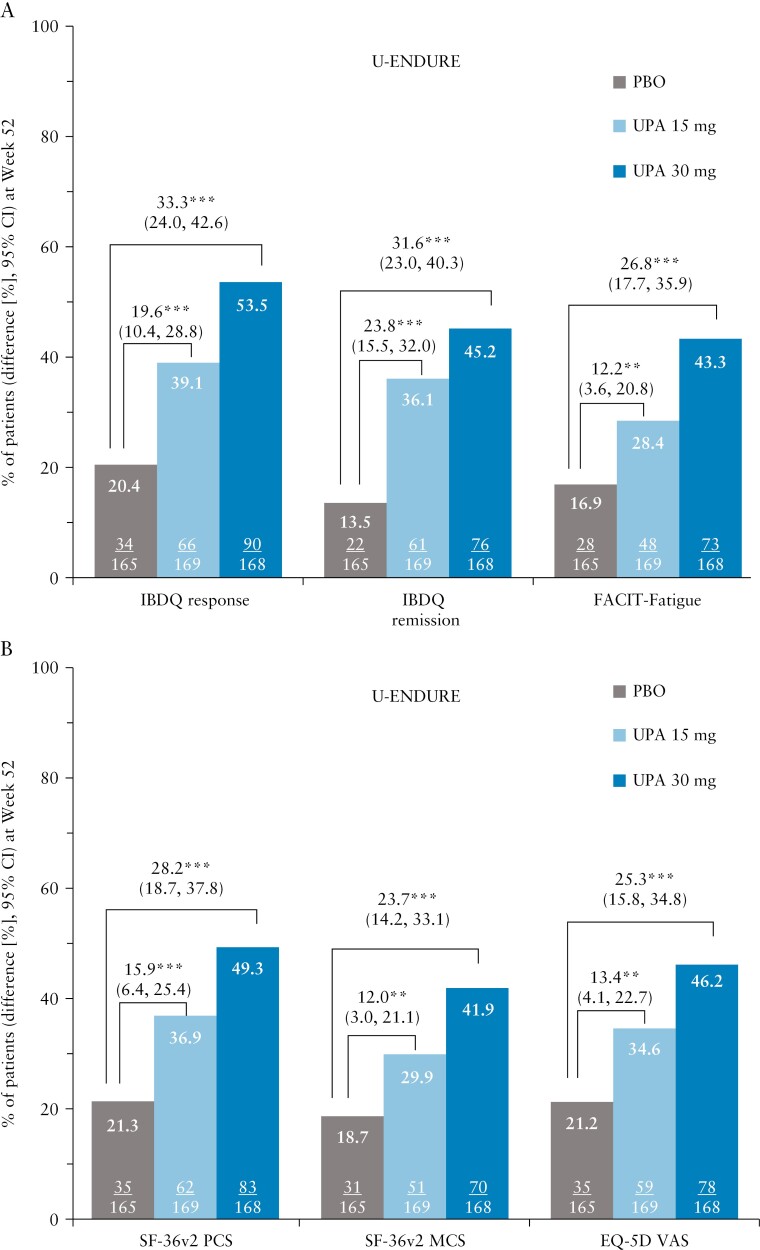
Percentage of patients reporting clinically meaningful improvements in [A] IBDQ response, IBDQ remission, FACIT-Fatigue, and [B] SF 36v2 PCS, SF-36v2 MCS, and EQ-5D at Week 52 of the U-ENDURE maintenance trial; *p ≤ 0.05; **p ≤ 0.01; ***p ≤ 0.001 for upadacitinib versus placebo. An increase of ≥ 16 points in IBDQ total score from baseline and a total score ≥ 170 points were considered IBDQ response and remission, respectively. An increase of ≥ 9-points in FACIT-Fatigue, ≥ 4.1-points in SF-36v2 PCS, ≥ 3.9-points SF-36v2-MCS, and ≥ 9.2-points in EQ-5D VAS from baseline was considered an MWPC. CI, confidence interval; EQ-5D VAS, EuroQol 5 Dimension Visual Analogue Scale; FACIT-Fatigue, Functional Assessment of Chronic Illness–Fatigue; IBDQ, Inflammatory Bowel Disease Questionnaire; MCS, Mental Component Summary; MWPC, meaningful within-person change; PBO, placebo; PCS, Physical Component Summary; SF-36v2, Short-Form Health Survey-36 version 2; UPA, upadacitinib.

In the U-EXCEL induction trial, a greater percentage of upadacitinib-treated patients compared with placebo had an IBDQ response [71.0% vs 50.2%; *p *≤ 0.001] and achieved IBDQ remission [44.2% vs 23.7%; *p *≤ 0.001] as early as Week 4 [Figure 1A]. The differences in IBDQ response and IBDQ remission between upadacitinib and placebo were sustained at induction Week 12 [Figure 1C]. Similar results were observed in the U-EXCEED induction trial [Figure 1B, D]. At maintenance Week 52 of U-ENDURE, a greater percentage of patients receiving upadacitinib 15 mg and 30 mg as maintenance treatment achieved an IBDQ response and IBDQ remission compared with placebo [IBDQ response: 39.1%, 53.5% vs 20.4%; IBDQ remission: 36.1%, 45.2% vs 13.5%, all *p *≤ 0.001; Figure 2A].

### 3.3. FACIT-Fatigue

Improvements in FACIT-Fatigue were observed as early as Week 4 of both induction trials with a greater percentage of upadacitinib-treated patients experiencing less fatigue than placebo [Figure 1A, B]. Between-group differences prevailed at Week 12 [Figure 1C, D]. At Week 52 of maintenance, a greater proportion of patients receiving upadacitinib 15 mg and 30 mg experienced improvement in FACIT-Fatigue compared with placebo [U-ENDURE: 28.4% and 43.3% vs 16.9%; *p *≤ 0.01; Figure 2A].

### 3.4. SF-36v2 PCS and MCS and EQ-5D VAS

At Week 4 of the induction trials, greater improvements in physical and mental function as assessed by SF-36v2 PCS and MCS, as well as general HRQoL measured by EQ-5D were observed in patients treated with upadacitinib 45 mg compared with placebo [proportions of patients with improvements ranging from 52.0–57.9% vs 37.2–45.8% for SF-36v2 PCS and MCS and EQ-5D, *p *< 0.05; Figure 1A, B]. Differences between upadacitinib and placebo for SF-36v2 PCS and MCS and EQ-5D VAS were even greater at Week 12 [Figure 1C, D]. At maintenance Week 52, approximately a third [29.9–36.9%] of patients treated with upadacitinib 15 mg and slightly less than half [41.9–49.3%] treated with 30 mg achieved clinically meaningful improvements in SF-36v2 PCS/MCS and EQ-5D compared with approximately one-fourth [18.7–21.3%] of patients in the placebo group [Figure 2B].

### 3.5. WPAI-CD domains

**Figure 3:**
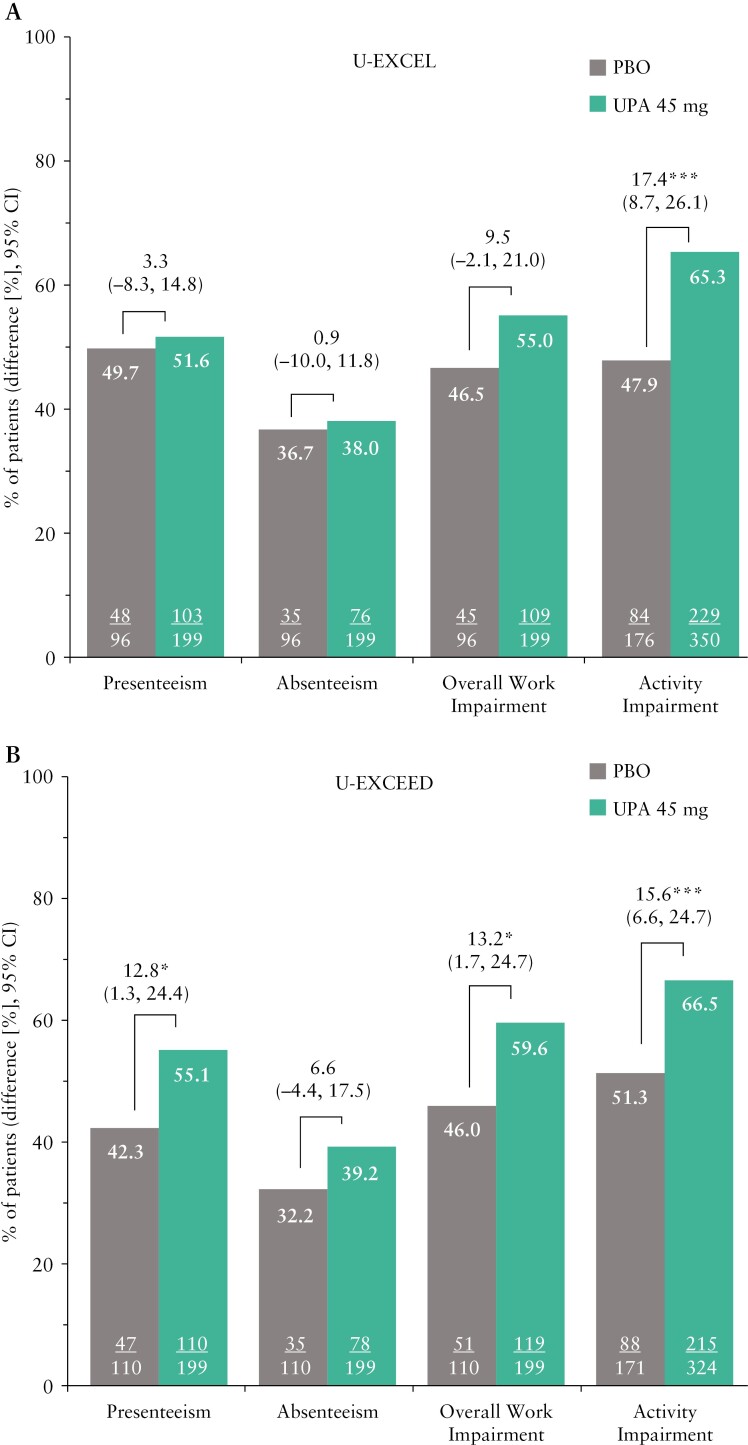

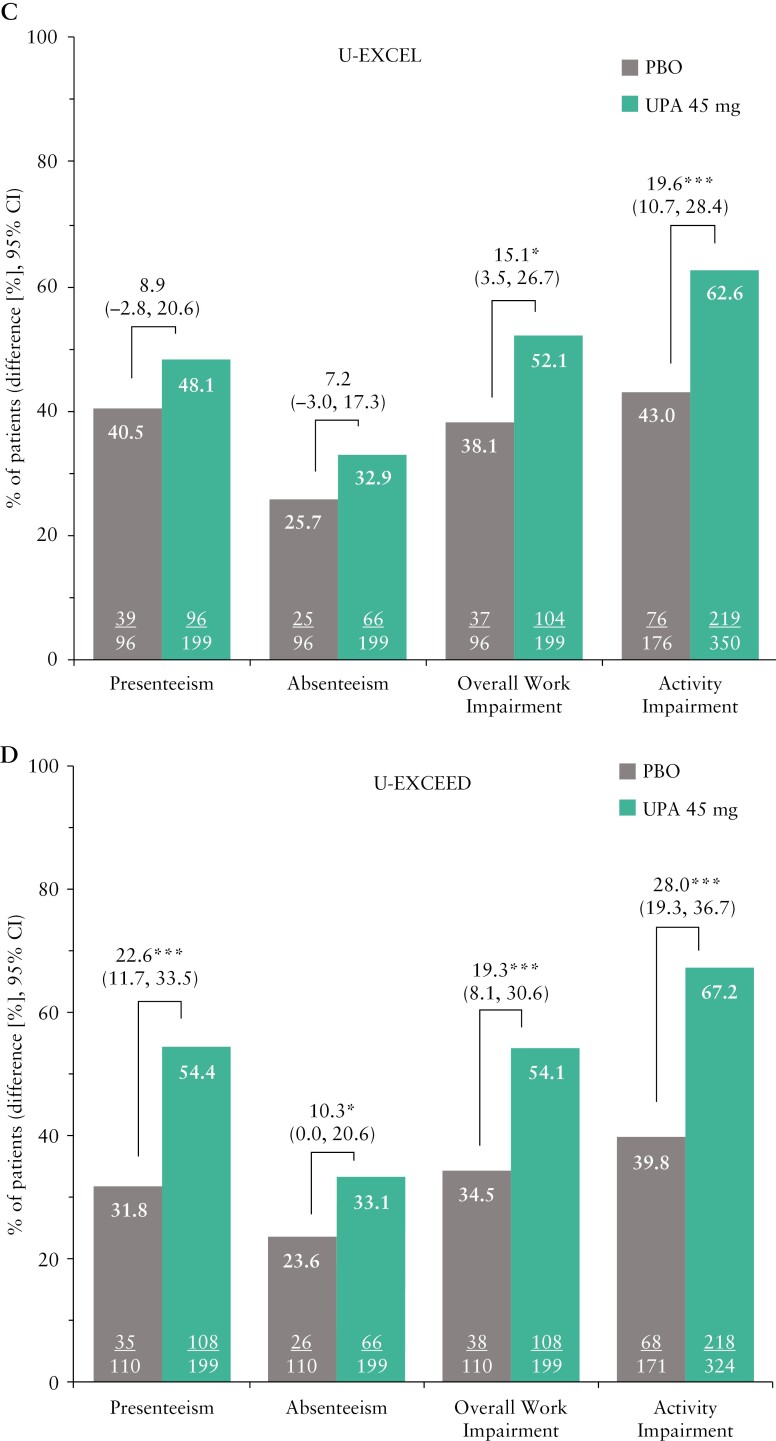
Percentage of patients reporting clinically meaningful improvements in WPAI-CD presenteeism, absenteeism, overall work impairment, and activity impairment at [A, B] Week 4 and [C, D] Week 12 of the U-EXCEL and U-EXCEED induction trials; *p ≤ 0.05; **p ≤ 0.01; ***p ≤ 0.001 for upadacitinib versus placebo. Presenteeism, absenteeism, and overall work impairment are reported only for patients who were employed at baseline. MWPC for presenteeism was defined as a ≥ 6.1% decrease from baseline. MWPC in absenteeism was defined as a ≥ 6.5% decrease from baseline. MWPC for activity impairment was defined as a ≥ 8.5% decrease from baseline. MWPC for overall work impairment response was defined as a ≥ 7.3% decrease from baseline. CI, confidence interval; MWPC, meaningful within-person change; PBO, placebo; UPA, upadacitinib; WPAI-CD, Work Productivity and Activity Impairment in Crohn’s Disease.

**Figure 4:**
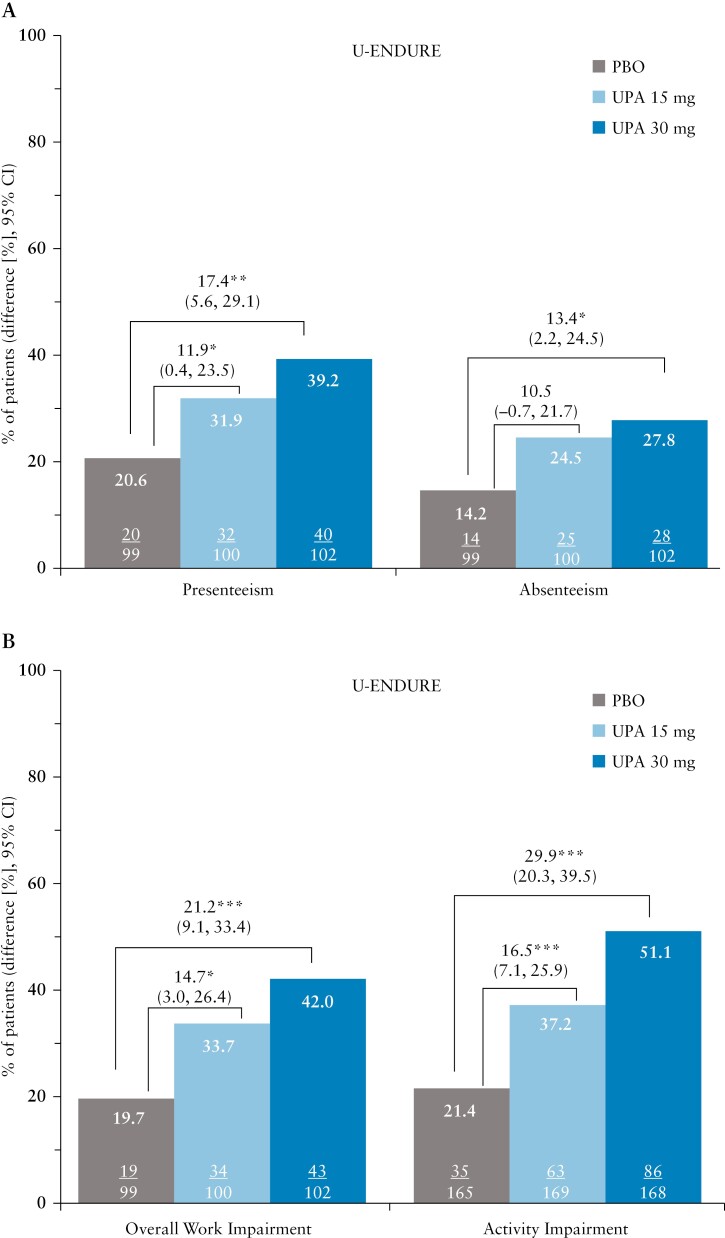
Percentage of patients reporting clinically meaningful improvements in WPAI-CD [A] presenteeism, absenteeism, and [B] overall work impairment, and activity impairment at Week 52 of the U-ENDURE maintenance trial; *p ≤ 0.05; **p ≤ 0.01; ***p ≤ 0.001 for upadacitinib versus placebo. Presenteeism, absenteeism, and overall work impairment data are reported only for patients who were employed at baseline. MWPC for presenteeism was defined as a ≥ 6.1% decrease from baseline. MWPC in absenteeism was defined as a ≥ 6.5% decrease from baseline. MWPC for activity impairment was defined as a ≥ 8.5% decrease from baseline. MWPC for overall work impairment was defined as a ≥ 7.3% decrease from baseline. CI, confidence interval; MWPC, meaningful within-person change; PBO, placebo; UPA, upadacitinib; WPAI-CD, Work Productivity and Activity Impairment in Crohn’s Disease.

The proportions of patients with clinically meaningful improvements in all four WPAI-CD domains were observed in patients treated with upadacitinib compared with placebo in the induction and maintenance trials [Figures 3 and 4]. Overall, the greatest improvements were observed in overall work impairment and activity impairment. At Week 4 of the induction trials, 55.0–59.6% of patients treated with upadacitinib 45 mg experienced clinically meaningful improvement in overall work impairment, compared with 46% in the placebo group [Figure 3A, B]. Similar improvements were noted at Week 12 [Figure 3C, D]. At Week 52, improvement in overall work impairment was greater in patients treated with upadacitinib 15 mg and 30 mg compared with placebo [33.7% and 42.0% vs 19.7%; *p *≤ 0.05; Figure 4B].

At Week 4, 65.3–66.5% of patients treated with upadacitinib 45 mg had a clinically meaningful improvement in their ability to perform daily activities, compared with 47.9–51.3% in the placebo group. At Week 52, improvement in activity impairment was sustained and greater in patients treated with upadacitinib 15 mg and 30 mg compared with placebo [37.2% and 51.1% vs 21.4%; both *p *≤ 0.001]. Dose-dependent responses were observed at Week 52 between upadacitinib 15 mg and 30 mg across all endpoints evaluated.

### 3.6. Relationship between achievement of clinical remission, endoscopic response, or corticosteroid-free remission and HRQoL measures

Generally, patients who achieved clinical remission, endoscopic response, or corticosteroid-free remission in the U-EXCEL and U-EXCEED induction trials also demonstrated improvements in HRQoL measures at greater rates, compared with those who did not achieve clinical remission, endoscopic response, or corticosteroid-free remission. At Week 12 in U-EXCEL, a greater percentage of patients who had an endoscopic response [vs those who did not] achieved an IBDQ response [72.5% vs 53.5%, *p *< 0.001] and IBDQ remission [54.4% vs 34.0%, *p *< 0.001], as well as clinically meaningful improvements in FACIT-Fatigue [53.3% vs 29.9%, *p *< 0.001], SF-36v2 PCS [71.4% vs 42.7%, *p *< 0.001], and MCS [58.2% vs 38.4%, *p *< 0.001, Supplementary Table 1]. Similar concurrent improvements in HRQoL outcomes were observed at Week 12 in patients who achieved clinical remission or corticosteroid-free remission versus those who did not [Supplementary Table 1]. A similar relationship between clinical remission, endoscopic response, or corticosteroid-free remission and HRQoL outcomes at Week 12 was observed in U-EXCEED [-Supplementary Table 2].

Examination of the relationship between FACIT-Fatigue and generic HRQoL [via SF-36v2 PCS/MCS] outcomes also demonstrated concurrent improvements at Week 12. In U-EXCEL, significantly more patients who demonstrated improvement in FACIT-Fatigue [vs those who did not] also achieved improvement in SF-36v2 PCS [87.0% vs 31.6%; *p *< 0.001]. A similar relationship was demonstrated for FACIT-Fatigue and SF-36v2 MCS [81.0% vs 23.3%, *p *< 0.001]. These results were also noted in U-EXCEED at Week 12, where patients who achieved clinically meaningful improvement in FACIT-Fatigue [vs those who did not] demonstrated concurrent improvement in both SF-36v2 PCS [86.7% vs 30.1%, *p *< 0.001] and SF-36v2 MCS [77.0% vs 21.4%, *p *< 0.001].

## 4. Discussion

For patients with moderately-to-severely active CD with one or more conventional and/or biologic therapy failure, upadacitinib 45 mg induction treatment improved fatigue and disease-specific and generic HRQoL measures compared with placebo. A greater proportion of patients treated with upadacitinib versus placebo achieved an IBDQ response, IBDQ remission, and clinically meaningful improvements in FACIT-Fatigue, SF-36v2 PCS and MCS, and EQ-5D as early as Week 4, which were generally sustained through Week 12. A higher percentage of patients receiving upadacitinib induction treatment achieved improvement in WPAI-CD presenteeism, absenteeism, and overall work impairment versus placebo in U-EXCEED, which included patients with a history of inadequate response or intolerance to one or more biologic therapies for CD. These findings suggest that upadacitinib may be a treatment option for patients who have previously failed other therapies. Furthermore, among patients who had a clinical response at Week 12 of induction, improvements in fatigue, HRQoL, and work productivity were sustained or enhanced through 52 weeks of maintenance treatment with upadacitinib 15 mg and upadacitinib 30 mg compared with placebo. Dose-dependent improvements in IBDQ response and remission, FACIT-Fatigue, SF-36v2 PCS and MCS, EQ-5D, and the WPAI-CD domains were observed.

Differences between upadacitinib and placebo groups in clinical remission were observed as early as Week 4 in both induction trials^25^; we can hypothesise that early clinical improvements along with tapering of corticosteroid use would parallel disease-specific [IBDQ response and remission] and generic [FACIT-Fatigue, SF-36v2 PCS, and SF-36v2 MCS] HRQoL improvements that are sustained with long-term treatment [U-ENDURE]. For example, when we analysed the relationship between achievement of clinical remission, endoscopic response, or corticosteroid-free remission and HRQoL measures at Week 12 of the induction studies, we found that a greater proportion of patients who achieved these outcomes [vs those that did not] had concurrent improvements in IBDQ response, IBDQ remission, FACIT-Fatigue, and SF-36v2 PCS and MCS. Furthermore, patients who reported clinically meaningful improvements in FACIT-Fatigue [relative to those who did not] at Week 12 also reported improvements in physical and mental function [via SF-36v2 PCS/MCS]. The early HRQoL improvements may be explained by the rapid action of Janus kinase inhibitors^46^ and their impact on disease symptoms.^47^

Improvements in IBDQ response, IBDQ remission, FACIT-Fatigue, SF-36v2 PCS, SF-36v2 MCS, and EQ-5D were consistent between the mixed study population in U-EXCEL [ie, patients with prior conventional or biologic therapy failure] and patients with prior biologic failures in U-EXCEED, indicating that upadacitinib treatment can lead to early HRQoL improvements irrespective of prior treatment failures. Consistent with the STRIDE-II recommendations, which highlight normalisation of quality of life as a core treatment goal,^2^ the onset of treatment response for HRQoL and fatigue, apparent as early as Week 4 of treatment, suggests that upadacitinib may enable patients with CD to establish and maintain a sense of normality shortly after initiating therapy.

Fatigue is the most common and burdensome systemic symptom reported by patients with CD.^23^ The overwhelming lack of energy or continuing tiredness that is not relieved^48^ has a negative impact on daily activities and often remains unaddressed during treatment due to prioritisation of clinical remission. The findings of this study indicate that patients receiving upadacitinib may experience early fatigue improvement at Week 4 of treatment. The sustained improvements in fatigue through Weeks 12 and 52 compared with placebo indicate that the effect observed in patients treated with upadacitinib is not subjective to a placebo effect. Additionally, limited understanding of the multifaceted nature of fatigue prevents health care professionals from addressing, assessing, or asking patients about fatigue, and in turn, prevents patients from reporting it and challenging fatigue as an inevitable symptom.^49^ Early improvements in fatigue may facilitate early doctor–patient discussions about common perceptions of fatigue, and enable monitoring or allaying patients’ relevant health concerns that affect their quality of life.^50^

Patients with CD experience significant physical [eg, poor sleep quality],^51^ emotional, and mental health challenges [eg, depressive symptoms, stress, anxiety],^52,53^ and are willing to trade a considerable part of their life expectancy for full recovery from the disease.^54^ These challenges are associated with worse quality of life and are partly explained by adverse illness perceptions resulting from patients’ thoughts of the chronic nature of the disease and having to live with uncontrollable symptoms.^52,55^ The early and sustained improvements in HRQoL and work productivity observed with upadacitinib may contribute to the modification of illness perceptions and more effective disease management, particularly when combined with referrals to psychological and/or social support.^52,56^ Considering the need to take a holistic view of patients’ health and HRQoL, including physical, emotional, and social aspects as well as their ability to work during clinical interventions,^2,57^ these findings are encouraging for patients receiving upadacitinib treatment.

The strengths of the U-EXCEL, U-EXCEED, and U-ENDURE trials include the employment of a wide array of patient-reported outcomes, such as IBDQ, FACIT-Fatigue, SF-36v2 PCS and MCS, EQ-5D, and WPAI-CD, which captured different aspects of disease-specific and generic HRQoL and work productivity in patients with CD receiving upadacitinib treatment. Additionally, patient data were collected and assessed longitudinally, including early time points, which enabled observing the onset and sustainability of HRQoL improvements. The randomised, double-blind, placebo-controlled design and the overall large sample size of the trials attest to the internal validity of the findings. The trials also had certain limitations, as the findings cannot be generalised to real-world settings and patients with milder CD who were not represented in the sample. Future trials investigating long-term HRQoL improvements beyond Week 52 of treatment with upadacitinib are warranted.

## Conclusion

In addition to the achievement of clinical remission and endoscopic improvements, the study findings indicate that upadacitinib improves disease-specific and generic HRQoL, fatigue, and work productivity in patients with CD with prior conventional or biologic treatment failure. HRQoL improvements in patients receiving upadacitinib were observed as early as Week 4 and sustained through 52 weeks of maintenance treatment. Future observational studies using real-world data are warranted to provide insight into long-term HRQoL-related benefits for patients with CD receiving upadacitinib treatment.

## Supplementary Data

Supplementary data are available at *ECCO-JCC* online.

jjae083_suppl_Supplementary_Data

## Data Sharing Statement

AbbVie is committed to responsible data sharing regarding the clinical trials we sponsor. This includes access to anonymised, individual, and trial-level data [analysis datasets], as well as other information [eg, protocols, clinical study reports, or analysis plans], as long as the trials are not part of an ongoing or planned regulatory submission. This includes requests for clinical trial data for unlicensed products and indications. These clinical trial data can be requested by any qualified researchers who engage in rigorous, independent, scientific research, and will be provided following review and approval of a research proposal, statistical analysis plan and execution of a Data Sharing Agreement. Data requests can be submitted at any time after approval in the USA and Europe and after acceptance of this manuscript for publication. The data will be accessible for 12 months, with possible extensions considered. For more information on the process or to submit a request, visit the following link: [https://vivli.org/ourmember/abbvie/] then select ‘Home’.
