# Supplementary material for: Impact of Upadacitinib Induction and Maintenance Therapy on Health-related Quality of Life, Fatigue, and Work Productivity in Patients with Moderately-to-severely Active Crohn’s Disease
Source: J Crohns Colitis. 2024 Jun 5;18(11):1804–18. doi: 10.1093/ecco-jcc/jjae083 (PMC11532615; doi:10.1093/ecco-jcc/jjae083)
Supplement: jjae083_suppl_Supplementary_Data [file jjae083_suppl_supplementary_data.pdf]

SUPPLEMENTARY DATA

Impact of upadacitinib induction and maintenance therapy on health-related quality of life, fatigue, and work productivity in patients with moderately-to-severely active Crohn's disease

Subrata Ghosh,[1] Brian G. Feagan,[2] Rogério Serafim Parra,[3] Susana Lopes,[4] Adam Steinlauf,[5] Yoichi Kakuta,[6] Namita Joshi,[7] Wan-Ju Lee,[7] Ana P. Lacerda,[7] Qian Zhou,[7] Si Xuan,[7] Kristina Kligys,[7] Nidhi Shukla,[7] Edouard Louis[8]

[1]College of Medicine and Health and APC Microbiome Ireland, University College Cork, Ireland; [2]Western University, London, ON, Canada; Alimentiv Inc. London, ON, Canada; [3]Ribeirão Preto Medical School, University of São Paulo, São Paulo, Brazil; [4]Centro Hospitalar e Universitário São João, Porto, Portugal; [5]IBD Clinical Center, Mount Sinai Hospital, New York, NY, USA; [6]Division of Gastroenterology, Tohoku University Graduate School of Medicine, Sendai, Japan; [7]AbbVie Inc., North Chicago, IL, USA; [8]Department of Gastroenterology, Centre Hospitalier Universitaire de Liège, Liège, Belgium

Supplementary Table 1. Relationship between CDAI clinical remission, endoscopic response, and corticosteroid-free clinical remission and patient-reported outcomes at Week 12: U-EXCEL.

	Patient-Reported Outcomes				
**n (%)**	**IBDQ Response**	**IBDQ Remission**	**FACIT-Fatigue**	**SF-36v2 PCS**	**SF-36v2 MCS**
**Clinical Remission**					
No	142/304 (46.7)	55/304 (18.1)	77/304 (25.3)	119/304 (39.1)	104/304 (34.2)
Yes	174/222 (78.4)***	161/222 (72.5)***	123/222 (55.4)***	158/222 (71.2)***	134/222 (60.4)***
**Endoscopic Response**					
No	184/344 (53.5)	117/344 (34.0)	103/344 (29.9)	147/344 (42.7)	132/344 (38.4)
Yes	132/182 (72.5)***	99/182 (54.4)***	97/182 (53.3)***	130/182 (71.4)***	106/182 (58.2)***
**Corticosteroid-free Clinical Remission**					
No	153/320 (47.8)	68/320 (21.3)	84/320 (26.3)	128/320 (40.0)	110/320 (34.4)
Yes	163/206 (79.1)***	148/206 (71.8)***	116/206 (56.3)***	149/206 (72.3)***	128/206 (62.1)***

***p < 0.001 for patients who achieved clinical remission, endoscopic response, or corticosteroid-free clinical remission versus those who did not. All missing values were considered non-responders. Clinical remission was defined as CDAI <150. Endoscopic response was defined as a decrease in SES-CD >50% from baseline of the induction study (or for subjects with an SES-CD of 4 at baseline, at least a 2-point reduction from baseline), as scored by central reviewer. Corticosteroid-free clinical remission was defined as discontinuation of corticosteroid use for CD and achievement of clinical remission per CDAI. IBDQ response was an increase of ≥16 points from

baseline, IBDQ remission was a total score ≥170 points, MWPC for FACIT-Fatigue was an increase of ≥9 points from baseline, MWPCs for SF-36v2 PCS and MCS were ≥4.1 and ≥3.9-point change from baseline, respectively.

CDAI, Crohn's Disease Activity Index; FACIT-Fatigue, Functional Assessment of Chronic Illness Therapy–Fatigue; IBDQ, Inflammatory Bowel Disease Questionnaire; MWPC, meaningful within-person change; MCS, Mental Component Summary; PCS, Physical Component Summary; SES-CD, simple endoscopic score for Crohn's disease; SF-36v2, Short-Form Health Survey-36, version 2.

Supplementary Table 2. Relationship between CDAI clinical remission, endoscopic response, and corticosteroid-free clinical remission and patient-reported outcomes at Week 12: U-EXCEED.

	Patient-Reported Outcomes				
**n (%)**	**IBDQ Response**	**IBDQ Remission**	**FACIT-Fatigue**	**SF-36v2 PCS**	**SF-36v2 MCS**
**Clinical Remission**					
No	137/334 (41.0)	63/334 (18.9)	93/334 (27.8)	128/334 (38.3)	106/334 (31.7)
Yes	145/161 (90.1)***	137/161 (85.1)***	103/161 (64.0)***	132/161 (82.0)***	109/161 (67.7)***
**Endoscopic Response**					
No	187/377 (49.6)	114/377 (30.2)	125/377 (33.2)	173/377 (45.9)	137/377 (36.3)
Yes	95/118 (80.5)***	86/118 (72.9)***	71/118 (60.2)***	87/118 (73.7)***	78/118 (66.1)***
**Corticosteroid-free Clinical Remission**					
No	147/345 (42.6)	73/345 (21.2)	102/345 (29.6)	137/345 (39.7)	114/345 (33.0)
Yes	135/150 (90.0)***	127/150 (84.7)***	94/150 (62.7)***	123/150 (82.0)***	101/150 (67.3)***

***$p < 0.001$ for patients who achieved clinical remission, endoscopic response, or corticosteroid-free clinical remission versus those who did not. All missing values were considered non-responders. Clinical remission was defined as CDAI <150. Endoscopic response was defined as a decrease in SES-CD >50% from baseline of the induction study (or for subjects with an SES-CD of 4 at baseline, at least a 2-point reduction from baseline), as scored by central reviewer. Corticosteroid-free clinical remission was defined as discontinuation of corticosteroid

use for CD and achievement of clinical remission per CDAI. IBDQ response was an increase of ≥16 points from baseline, IBDQ remission was a total score ≥170 points, MWPC for FACIT-Fatigue was an increase of ≥9 points from baseline. MWPC for SF-36v2 PCS was ≥4.1-point change from baseline and for SF-36v2 MCS was ≥3.9-point change from baseline.

CDAI, Crohn’s Disease Activity Index; FACIT-Fatigue, Functional Assessment of Chronic Illness Therapy–Fatigue; IBDQ, Inflammatory Bowel Disease Questionnaire; MWPC, meaningful within-person change; MCS, Mental Component Summary; PCS, Physical Component Summary; SES-CD, simple endoscopic score for Crohn’s disease; SF-36v2, Short-Form Health Survey-36, version 2.
